# On the Use of Arsenic in Psoriasis

**Published:** 1807-03

**Authors:** 


### To Dr. BATTY.

Dear Sir,

As you suggested the remedy which has effectually cured me of a very disagreeable complaint, you, perhaps, may wish to have a few particulars of the Case; and if you think the following account deserves a corner of the Medical Journal, I have not the smallest objection to your making what use of it you please.

It is now above a year since I shewed you my hands, wrists, and ankles; you must recollect the deplorable state they were in. I am happy to tell you they were cured in about five weeks, and have remained quite well ever since. The complaint began so long ago as the year 1800, with a violent itching above the pubis, which subsided, and returned with more or less violence at intervals; it was generally alleviated by scratching, which separated a few branny scales; however, as the itching was only occasionally troublesome, no particular remedy was applied beyond cold, and sometimes by way of experiment, hot water, neither of which did any service: it declined altogether in about three months.—On its disappearance, the palm of my right hand became thickened, hard, dry, and inclined to part with its cuticle; this also itched violently, and became scaly, the skin cracked and left deep fissures, very sore and painful on stretching the fingers; it spread gradually up to the wrist, which became as sore and unpleasant as the hand.—I now began to think the complaint of sufficient consequence to require advice, and during the space of eight or nine months, I tried every medicine and application that was ordered, but without the least effect; I need not transcribe the exact formulæ, but I used ointments with calomel, white præcipitate, red præcipitate, ung. hydrargyri nitrati, solutions of sublimate, and diluted lunar caustic, tar ointments and sulphur, and a variety of internal medicines.

It is necessary to state to you, that my general health was excellent, and if it had not been for this vexatious wrist, I never enjoyed society, dinners, and conviviality with more relish. When the wrist and hand were at the worst, the palm of the left hand chose to follow the example of the right; and together with the left wrist became as sore and uncomfortable as the other. My right and left inner ankles also soon shewed the same unhappy disposition, and these latter were afterwards infinitely more painful and troublesome than the wrists. The constant itching, scratching, and subsequent smarting inflamed me, and disturbed my rest. I was under the necessity of losing a little blood, lowering my diet, and abstaining totally from wine. This plan only served to pull down my strength, without abating one *item* of the complaint. Its severity induced me to examine every author who had written on cutaneous diseases, and it seemed to come nearest to Dr. Willan's description of *Psoriasis diffusa,* and *palmaria,* of any thing I found, only with this difference, not being accompanied with any constitutional affection, which he says invariably is the case with *Psoriasis.* However, if the descriptions had tallied in every point, I should have been no gainer by it. I found no relief from any of the means he recommends. Perhaps I ought to be more particular in detailing the remedies I took. Altho' I had not the least venereal affection, I thought it right to take mercurials as alteratives, and rub in the ung. hydrarg. fort. till it kept my gums sore for a month. These affected my constitution, in as much as they weakened my strength and impaired my appetite, without procuring the least alteration in the palms, wrists, and ankles. I drank the Harrowgate-water, and also took the sulphur smelling *Bit noben,* an Indian remedy of some celebrity; the diluted nitric acid was taken a long time without effect, and also soda in pills. I tried hot and cold baths both fresh and salt, and was equally unfortunate. One thing I observed, that, although the complaint was nearly stationary, the itching was more intense when my mind was not actively employed.—This itching I occasionally averted by absorbing myself, in an interesting novel and books of entertainment; but these soon lost their effect, and I found myself obliged to study mathematical problems and algebraical calculations with all the eagerness I could muster. The complaint appeared at times as if disposed to abate in violence; and as it had lasted some years, I endeavoured to refer its remissions to particular seasons, but this did not turn out true, above once or twice, and therefore must have been accidental.

When I applied to you, you expressed your surprise at my neglecting to try arsenic, as it had been so respectably recommended by Dr. Girdlestone of Yarmouth, in the Medical Journal, and of which I was a constant reader.

I began its use without delay, and in less than a fortnight, the scales on the wrists and ankles became much smaller, smaller, and rubbed off like powder; the palms of my hands first recovered their proper texture, and in less than six weeks I was quite well, and discontinued the arsenic. The solution of arsenic I procured at Apothecaries Hall, and I began with three drops twice a day in a little water, and gradually increased the dose till I took seven drops twice a day; if I exceeded this number, my bowels were affected, and as I found I was curing, I adhered to the seven drops. In my opinion, this is an excellent instance of the powerful effect of one remedy; it was tried alone; it had no co-operation of regimen, nor applications to the parts. I was not under the influence of a milk and vegetable system. I was not *sweetening my blood* with decoctions of elm bark, mesereon, sassafras, and of sarsaparilla, which I took in considerable quantity after the mercurial course; and my health, which I am certain was uniformly good, except when disturbed by physic and scratching, is now as good as ever it was.

I have been lately informed that the Italian slow poison, called *Acquetta,* or *Aqua Tuffana,* is a weak solution of arsenic, and that the Italians can calculate to a day when it will kill. If this is true, I have either not taken a sufficient dose, or, as Mother Cole says, "my time is not yet come." I have no fears about it, nor do I think your readers need have any hesitation in recommending it in cases similar to mine.

I am, &c.

